# Supplementary material for: Spontaneous calcium transients in hair cell stereocilia
Source: Sci Rep. 2025 Sep 29;15:33660. doi: 10.1038/s41598-025-17976-1 (PMC12480455; doi:10.1038/s41598-025-17976-1)
Supplement: Supplementary file 1 — Supplementary Material 1 [file 41598_2025_17976_MOESM1_ESM.docx]

**Movie Legends**

**Movie S1: Spontaneous calcium transients occur in individual stereocilia.**

Shown here are P5 mouse inner hair cells visualized with membrane-localized GCaMP3. The area marked by a white rectangle is zoomed in on the right. A calcium transient spanning 5 s can be observed in an individual stereocilium in this region. Frames are 1 s apart. Scale bar is 1 µm

**Movie S2: Stereocilia in various type of hair cells exhibit spontaneous calcium transients.**

Examples of calcium transients are shown in P4 inner hair cells (*top left*, Frame interval 700 ms, Scale bar 2 µm), P4 utricular hair cell (*top right*, Frame interval 1 s, Scale bar 3 µm), P10 outer hair cells (*bottom left*, Frame interval 500 ms, Scale bar 3 µm) and P3 saccular hair cells (*bottom right*, Frame interval 500 ms, Scale bar 5 µm).

**Movie S3: Treatment with the MET channel blocker Amiloride blocks calcium transients in stereocilia.**

P6 sacculus hair cells were treated with 50 µM Amiloride and imaged every 400 ms. Compared to the Control (*left*), the Amiloride-treated stereocilia (*right*) exhibit significantly reduced calcium activity. Scale bar is 5 µm.

**Movie S4: Spontaneous calcium transients are observed in unexpected locations.**

*First movie –* A P5 organ of Corti inner hair cell exhibits a calcium transient in a stereocilium of the tallest row (white arrow), where the MET channel is not believed to be present. Frames are 600 ms apart.

*Second movie* – A P9 saccular hair bundle is shown with a stereocilium exhibiting a calcium transient that originated from the base (white arrow), rather than the tip. Frames are 600 ms apart.

*Third movie –* A P4 utricle hair bundle is shown with a calcium transient originating from the middle of a stereocilium (white arrow). Frames are 300 ms apart.

All scale bars are 5 µm.

**Movie S5: Calcium transients also occur in microvilli-like stereocilia precursors.**

A developing P4 inner hair cell is shown with its apical surface covered with microvilli-like stereocilia precursors. Infrequent calcium transients can be seen in these microvilli
(white arrows). Frames are 1 s apart. Scale bar is 1 µm.

**Movie S6: Hair cell bodies exhibit spatiotemporally uncorrelated spontaneous calcium activity.**

A P3 organ of Corti is shown with inner hair cells exhibiting spontaneous activity that is not correlated between neighboring cells. The activity is visualized by cytosolic GCaMP6f. Frames are 500 ms apart. Scale bar is 5 µm.

**Movie S7: Spontaneous calcium transients in stereocilia occur in hair cells with cell body activity.**

Shown here are P7 ampullar hair cells exhibiting spontaneous calcium transients in stereocilia as well as occasional hair cell body activity. A depth of 8.8 µm (11 z slices, 0.8 µm slice interval) was imaged every 2 s. Ampullar tissue was mounted at an angle that enable the visualization of the entire cell body as well as the bundle in the same plane. Scale bar is 5 µm.

**Movie S8: Membrane tethers in over deflected hair bundles can generate calcium transients from their tips.**

When hair bundles are over-deflected, they form membrane tethers between stereocilia (*left*, white arrow) and between a stereocilium and the coverslip (*right*, white arrow). Calcium transients can originate from the tips of these membrane tethers (white arrows). Frames are 1 s apart. Scale bar is 5 µm.

**Movie S9: Spontaneous calcium transients occur *in vivo* in zebrafish hair cells.**

Spontaneous calcium transients in individual stereocilia occur *in vivo* as seen in zebrafish inner ear hair cells at 3 dpf (*left*), in developing posterior lateral line hair cells at 2 dpf (*middle*), and in predominantly mature posterior lateral line hair cells at 5 dpf (*right*). Frames are 5 s apart. Scale bar is 2 µm.

**Movie S10: Spontaneous calcium activity continues to occur in the organ of Corti inner hair cells after the onset of hearing.**

Shown here are organ of Corti inner hair cells (IHCs) in an acute tissue preparation at P17. Spontaneous activity continues to occur in the stereocilia and can be seen in the tallest row of stereocilia (white arrows). Frames are 200 ms apart. Scale bar is 5 µm.
