# Supplementary material for: Spontaneous calcium transients in hair cell stereocilia
Source: Sci Rep. 2025 Sep 29;15:33660. doi: 10.1038/s41598-025-17976-1 (PMC12480455; doi:10.1038/s41598-025-17976-1)
Supplement: Supplementary file 4 — Supplementary Material 4 [file 41598_2025_17976_MOESM4_ESM.pdf]

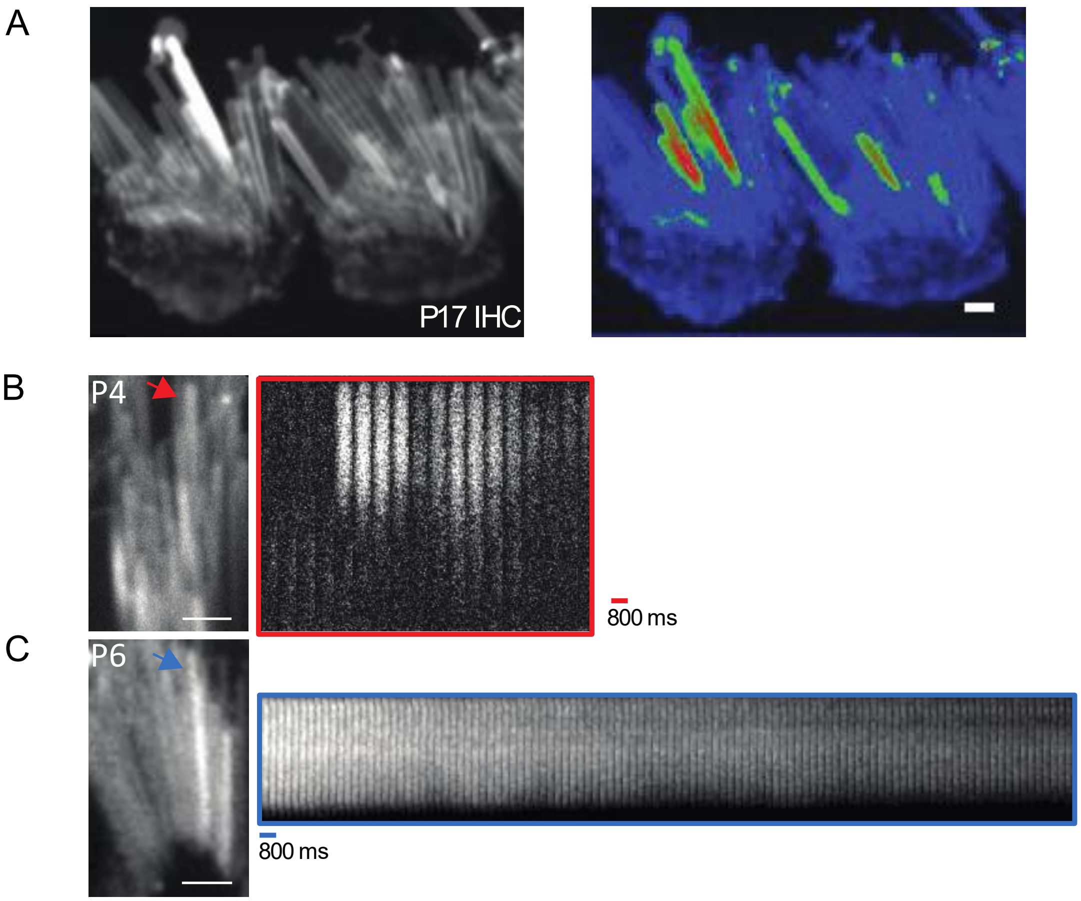

Supplementary Figure 1: Individual calcium transients have variable rise and decay times and continue after the onset of hearing.

A) Imaging membrane-localized GCaMP3 in acute tissue explants of the organ of Corti at P17 shows that these transients continue after the onset of hearing and MET. The left panel shows inner hair cells at P17 that were imaged at a time interval of 200 ms. The right panel shows the standard deviation projection of the timelapse, where calcium activity in individual stereocilia can be observed. **B)** A P4 utricle is shown with a kymograph of the stereocilium marked with a red arrow. The calcium transient peaks within a time frame of 800 ms. **C)** An example of a

transient with a slow decay. Shown here is a P6 sacculus with a kymograph of the stereocilium marked with a blue arrow. Scale bars for all panels are 1 μm.

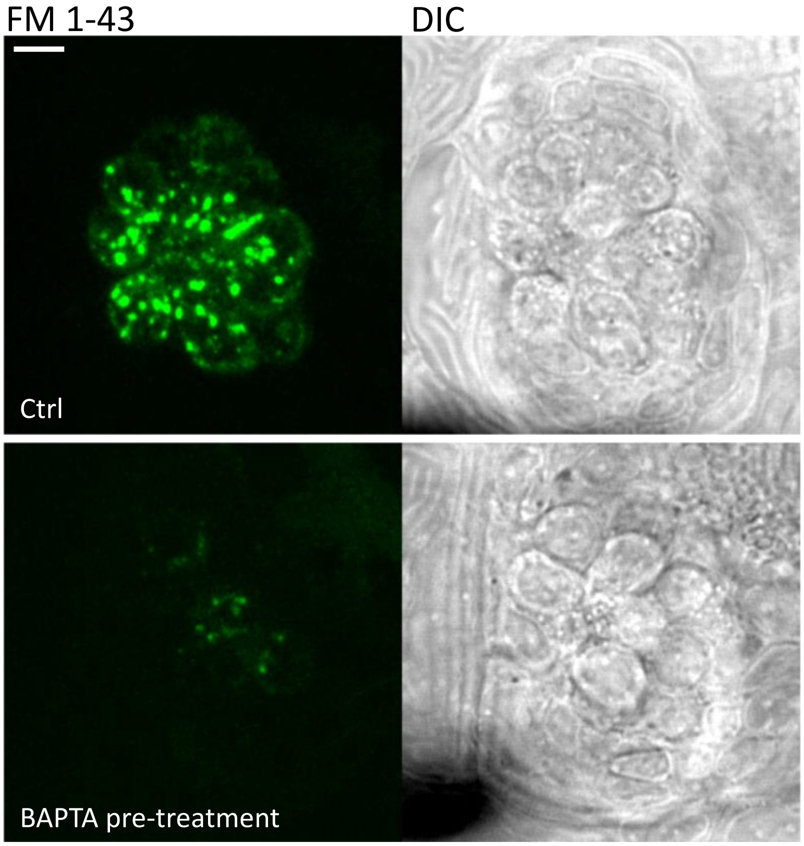

Supplementary Figure 2: Pre-treatment with BAPTA results in reduced FM1-43 uptake

In order to determine whether the tip links were successfully broken with our BAPTA pre-treatment (5 mM for 15 min), we tested hair cell function using the dye FM1-43, which enters the hair cells through the MET channel. When tip links are broken, the dye uptake is greatly reduced as can be visualized in the images. Shown here are wild type and BAPTA pre-treated neuromasts at 5 days post fertilization imaged with identical settings. We imaged 3 control and 2 BAPTA pre-treated neuromasts in this experiment. Scale bar is 5 μm.
